# An Updated View on the Antiviral Therapy of Hepatitis C in Chronic Kidney Disease

**DOI:** 10.3390/pathogens10111381

**Published:** 2021-10-26

**Authors:** Fabrizio Fabrizi, Roberta Cerutti, Piergiorgio Messa

**Affiliations:** 1 Division of Nephrology, Dialysis, and Kidney Transplant, Ca’ Granda IRCCS Foundation and Maggiore Policlinico Hospital, 20122 Milano, Italy; Roberta.cerutti@policlinico.mi.it (R.C.); piergiorgio.messa@policlinico.mi.it (P.M.); 2 Department of Clinical Sciences and Community Health, University of Milan, 20122 Milan, Italy

**Keywords:** dialysis, direct-acting antiviral agents, Hepatitis C virus, sustained viral response

## Abstract

Background: Hepatitis C virus infection remains common in patients with chronic kidney disease, including those on maintenance dialysis. The relationship between hepatitis C virus infection and chronic kidney disease is bi-directional; in fact, HCV is both a cause and consequence of chronic kidney disease. According to a systematic review with meta-analysis of observational studies (*n* = 23 studies) (*n* = 574,081 patients on long-term dialysis), anti-HCV positive serologic status was an independent and significant risk factor for death in patients with advanced chronic kidney disease on long-term dialysis. The overall estimate for adjusted mortality (all-cause death risk) with HCV was 1.26 (95% CI, 1.18; 1.34) (*p *< 0.0001). Interferon-based therapies are biased by low efficacy/safety in chronic kidney disease, but the advent of direct-acting antiviral drugs has made a paradigm shift in the treatment of HCV-infection. These medications give interruption of viral replication because they target specific non-structural viral proteins; four classes of DAAs exist-NS3/4A protease inhibitors, NS5A inhibitors, NS5B nucleoside and non-nucleoside polymerase inhibitors. All-oral, interferon-free, ribavirin-free combinations of DAAs are now available. Aim: The goal of this narrative review is to report the available treatment options for HCV in advanced chronic kidney disease. Methods: We have made an extensive review of the medical literature and various research engines have been adopted. Results: Some combinations of DAAs are currently recommended for HCV in advanced CKD (including patients on maintenance dialysis): elbasvir/grazoprevir; glecaprevir/pibrentasvir; and sofosbuvir-based regimens. Solid evidence, based on registration and “real life” studies supports their efficacy (SVR rates > 90%) and safety even in patients with advanced CKD. No dosage adjustment is necessary and treatment duration is 8–12 weeks. However, recent data highlight that many patients with advanced CKD remain untreated, and numerous barriers to antiviral treatment of HCV still exist. Whether successful antiviral therapy with DAAs will translate into improved survival in the advanced CKD population is another point of future research.

## 1. Introduction 

Patients with chronic kidney disease, particularly those who undergo regular dialysis and kidney transplant recipients, are frequently infected with the hepatitis C virus, which is an important cause of mortality in this population [1]. Various reports have reported a reduction of frequency rates of HCV infection within dialysis units during the last decade, but transmission of HCV infection between patients on regular haemodialysis continues to occur globally [2]. The recent introduction in the market of direct-acting antiviral agents is dramatically changing the management of hepatitis C in both patients with intact kidneys and in those with chronic kidney disease. The antiviral therapy of HCV has been advocated by some authors as an additional option to control HCV within haemodialysis units [3,4]. The aim of this narrative review is to give information on the antiviral treatment of hepatitis C virus in patients with advanced CKD (stage 4–5 CKD), and we report here the most recent advances in this field.

## 2. Information Sources and Search Strategy

Studies were identified by searching electronic databases and sources of gray literature. The literature search was applied to PubMed MEDLINE, EMBASE, and Google Scholar. The following keywords were adopted: (”Hepatitis C Virus” OR “HCV” OR “Hepatitis C”) AND (“chronic kidney disease” OR “End-stage kidney disease” OR “Renal Insufficiency” OR “Renal impairment”) AND (“Interferon” OR “Ribavirin” OR “Direct-acting antiviral agents” OR “Sofosbuvir” OR “Antiviral therapy”). We have considered published articles from 1 January 2010, to 31 July 2021. Only English language articles were included.

## 3. Current Epidemiology of HCV in Chronic Kidney Disease

After its identification in 1989, it has been observed that patients on renal replacement therapy have commonly detectable anti-HCV antibody in serum. The frequency of patients with positive serology for anti-HCV antibody who undergo maintenance dialysis is still higher than that observed in the respective general population of developed [2] or emerging countries [2,5,6,7,8,9,10,11,12,13,14,15]. Infection with chronic HCV infection leads to chronic liver disease with its attendant complications (cirrhosis, hepatocellular carcinoma, and hepatocellular failure). A large body of studies, published in the 1990s, concerned epidemiology and risk factors for HCV infection among patients undergoing haemodialysis, particularly in the developed world. More recently, the medical literature regarding the epidemiology of HCV in dialysis has been sparse.

The Dialysis Outcomes and Practice Patterns Study (DOPPS, 1996–2015) was recently published; it has given us deeper insight on this topic [2]. According to the latest survey from the DOPPS, the prevalence of anti-HCV antibody in patients on HD ranged from 4.1% (Belgium) to 20.1% (Gulf Cooperation Council Countries). Prevalence of anti-HCV antibody decreased during the past 15 years in 5 countries that had participated in the DOPPS since phase 1 including Spain (dropped from around 21% to 9%), and France (dropped from around 14% to 9%). In some countries (Germany), prevalence remained stable. The DOPPS investigators found that the incidence of HCV infection decreased from 2.9 to 1.2 per 100 patient-years in countries participating in the initial phase of the study. The most important predictors for de novo HCV were facility HCV prevalence (HR, 1.95, 95% CI, 1.44–2.65 in facilities with HCV prevalence > 20%), HBV (HR, 2.87, 95% CI, 2.06–4.00), HIV infection 2.93 (95% CI, 1.79–4.80), and time on dialysis (HR, 1.36, 95% CI, 1.07–1.73 for 10 years or more) [2].

Information on the prevalence and incidence rates of HCV among patients on regular haemodialysis in the emerging world is less abundant; however, numerous single-centre surveys from emerging countries have been published in the last decade and these have reported prevalence rates of up to 80.7% [2,5,6,7,8,9,10,11,12,13,14,15].

## 4. Natural History of HCV in Chronic Kidney Disease

It is not easy to provide a detailed evaluation of the natural history of HCV infection in patients with advanced chronic kidney disease, particularly those on maintenance dialysis. Various reasons exist to explain this—the natural history of HCV spans usually decades in patients with intact kidneys, whereas dialysis patients have limited life expectancy. In fact, patients with chronic kidney disease have higher morbidity and mortality than the general population, due to aging and comorbidities. HCV infection is frequently asymptomatic with an apparently indolent course even in patients with advanced chronic kidney disease. Aminotransferase levels are lower in patients on maintenance dialysis; thus, it is difficult to recognise the occurrence of liver disease on the grounds of biochemical abnormalities. 394 patients undergoing regular haemodialysis in the greater Los Angeles area were retrospectively evaluated by anti-HCV ELISA2 and branched-chain DNA assay for detection of anti-HCV antibody and HCV RNA in serum. Serum transaminase values were greater in HCV RNA positive than in HCV RNA-negative patients, 23.8 (95% CI, 60.8–9.3) vs. 17.1 (95%CI, 50.4–5.8) IU/L (*p* = 0.009) and 14.4 (95% CI, 48.9–4.3) vs. 9.8 (95% CI, 37.3–2.5) IU/L (*p* = 0.008) [16]. According to logistic regression analysis, HCV viremia was linked to positive anti-HCV serologic status (*p* = 0.0001) and ALT activity (*p* = 0.01) [16]. The current availability of direct-acting antiviral agents, which are provided of great efficacy and safety, precludes the implementation of observational studies with large size and prolonged follow-up to analyse the course of chronic HCV infection in end-stage kidney disease.

It has been stated that survival in most patients with CKD stage 1 and 2 is not different from that observed in the general population with intact kidneys. Survival in patients with CKD stage 3–5 is lower than that observed in the general population, and some information has been recently accumulated on the link between positive anti-HCV serologic status and survival in the dialysis population. Death can be considered a reliable endpoint in the context of observational studies assessing the course of HCV over time in patients with intact kidneys or end-stage kidney disease and some clinical studies have been carried out to this aim. We have recently made a systematic review with meta-analysis of observational studies (*n* = 23 studies) (*n* = 574, 081 patients on long-term dialysis). We found that positive anti-HCV serologic status was an independent and significant risk factor for death in the dialysis population. The overall estimate for adjusted mortality (all-cause death risk) with HCV was 1.26 (95% CI, 1.18; 1.34) (*p *< 0.0001) [1]. We performed stratified analyses to understand the cause of the increased death risk, the summary estimate for adjusted mortality (liver disease-related mortality) was 5.05 (95% CI, 2.53–10.0) (*p *< 0.0001). The pooled estimate for cardiovascular death risk was 1.18 (95% CI, 1.085–1.29) (*p *< 0.0001). According to our meta-regression analysis, the relationship between positive anti-HCV serologic status and all-cause death risk was higher in studies provided with greater size (*p *< 0.0001), greater frequency of diabetics (*p* = 0.0005) and HCV-positive individuals (*p* = 0.001) [1].

## 5. Antiviral Therapy of HCV and Its Aim

The goal of antiviral therapy of HCV is to ‘cure’ infection and the endpoint of therapy is the achievement of SVR12, i.e., the clearance of HCV RNA from serum which persists at least 12 weeks after the end of antiviral therapy. Some evidence exists showing that SVR occurrence is associated with improved survival and better quality of life. Treatment is recommended for all patients with HCV infection (HCV RNA positive patients) without contraindications for treatment. Priority should be given to various patient groups, including those with advanced fibrosis and/or cirrhosis and those with advanced CKD.

## 6. Antiviral Therapy of HCV in Dialysis Patients (IFN-Based Therapy)

The recent introduction in the market of DAAs for the treatment of HCV has dramatically changed the management of HCV, even in patients with advanced chronic kidney disease. IFN-based therapies have limited efficacy and safety, particularly in patients with end-stage kidney disease; thus, clinicians have been reluctant to provide antiviral therapy to these patients and most patients with HCV infection and chronic kidney disease have not been treated so far. Patients with HCV and end-stage kidney disease have been historically considered a “difficult-to-treat” patient group, as well as patients with HIV/HCV or HBV/HCV co-infection, among others [17].

The scientific literature on monotherapy with conventional or pegylated interferon for chronic HCV in the dialysis population is not abundant, and it is mostly based on clinical studies provided with small size. As mentioned above, this is probably due to the reluctance of clinicians in treating viral hepatitis C in patients with chronic kidney disease. Two randomized controlled studies have suggested that combined antiviral therapy (peginterferon plus low-dose ribavirin) provides greater SVR rates compared with peginterferon monotherapy alone [18,19].

We carried out a systematic review of the literature with a meta-analysis of clinical studies to address the efficacy and safety of monotherapy with peg-IFN in dialysis population. We retrieved twenty-four clinical studies (*n* = 744 unique patients); five studies were RCTs. The overall estimate for sustained viral response and drop-out rate was 0.40 (95% CI, 0.35; 0.46) and 0.14 (95% CI, 0.09; 0.20), respectively. The most frequent side-effects requiring discontinuation of treatment were haematological (31/83 = 37%) and gastrointestinal (9/31 = 10.8%) [20]. The medical literature concerning combined antiviral therapy (conventional or pegylated interferon plus ribavirin) for HCV in patients on maintenance dialysis is even more sparse. On the basis of a systematic review of the literature, we made a meta-analysis of clinical studies with the goal to evaluate the efficacy and safety of antiviral therapy with peg-IFN plus ribavirin in dialysis population. We identified eleven clinical studies (*n* = 287 unique patients), two of which were controlled clinical trials. The summary estimate for SVR and drop-out rate were 0.60 (95% CI, 0.47; 0.71) and 0.18 (95% CI, 0.08; 0.35), respectively. The most common sources of dropouts were anaemia (11/46 = 23%) and infections (6/46 = 13%) [21]. Haemolytic anaemia induced by ribavirin is frequent among patients with stage 4–5 CKD and can be severe despite early use of ESAs. This is related to the ribavirin accumulation in red blood cells and the poor clearance of the drug by the haemodialysis procedure.

In short, these meta-analyses have shown that the IFN-based therapies have limited efficacy and safety in dialysis population. The Kidney Disease: Improving Global Outcomes (KDIGO) HCV Study Group recommended low-dose ribavirin (200 mg day) in patients with end-stage kidney disease in order to avoid haemolytic anaemia due to ribavirin accumulation in patients who are already anaemic at baseline [4].

## 7. Antiviral Therapy of HCV in Patients with Advanced CKD (DAAs)

**Figure 1:**
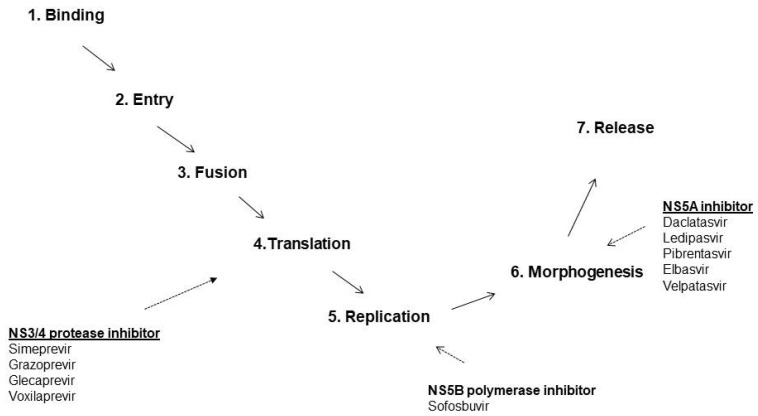
Replication of HCV Genome and direct-acting antiviral agents with respective targets.

**Figure 2:**
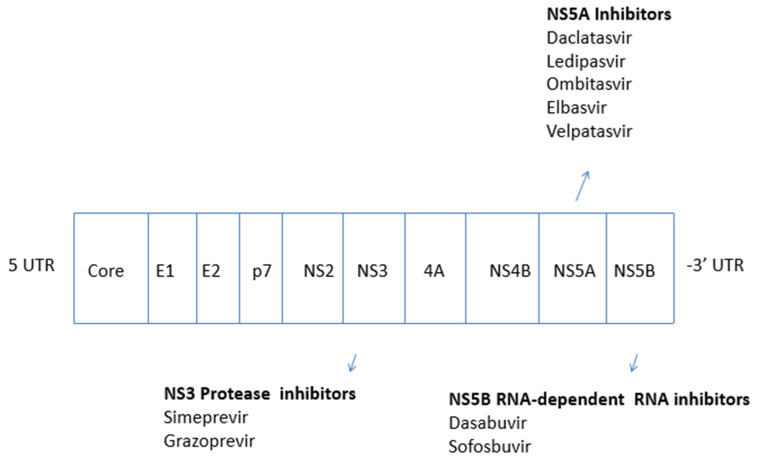
Genome of HCV and DAAs.

All-oral, sofosbuvir-based, interferon-free, combinations of DAAs were approved in 2014. Four classes of DAAs that target specific non-structural proteins of the HCV genome and result in the interruption of replication of HCV are now available on the market (Figure 1). These are defined by their mechanism of action and therapeutic target—NS3/4A protease inhibitors, NS5B nucleoside polymerase inhibitors, NS5B-non nucleoside polymerase inhibitors, and NS5A inhibitors (Table 1) (Figure 2).

According to an observational multicentre survey performed in patients on haemodialysis (period 2012–2015), the number of patients on HD who received antiviral therapy was extremely small- 80 of 5313 HCV positive patients on HD who had prescription data. Prescription of DAAs was defined by the authors as “particularly rare”, only 11 patients (*n* = 7 from the US, *n* = 1 from Australia, Canada, France, Sweden) underwent antiviral therapy with DAAs (period 2012–2016) [22].

DAAs have modified treatment paradigms for HCV, offering shorter, well-tolerated and very effective therapies. Regimens with DAAs now represent the standard of care for acute or chronic HCV in patients with intact kidneys. A few combinations of DAAs are currently recommended for antiviral treatment of HCV in patients with CKD stage 4 or 5, regardless they were dialysis-dependent or not.

## 8. PI Containing SOF-Free DAAs (Clinical Trials)

The C-SURFER (Hepatitis C: Study to Understand Renal Failure’s Effect on Responses) was the first registration trial of the use of PI containing SOF-free DAAs in patients with advanced CKD (Table 2). It is a phase 3 study on all-oral, IFN-free and ribavirin-free regimen for treatment of HCV in patients with advanced CKD [23,24]. Patients were randomised to receive grazoprevir (NS3/4A protease inhibitor) and elbasvir (NS5A inhibitor) (immediate treatment group) or placebo (deferred treatment group) 100 mg GRZ/50 mg EBR once daily for 12 weeks. At week 16, patients of deferred treatment group underwent therapy with GRZ/EBR. There were three study groups—immediate (*n* = 111) and deferred treatment (*n* = 113), and intensive pharmacokinetic cohort (*n* = 11). Modified intention-to-treat (m ITT) SVR12 rate in the cohort including immediate treatment and pharmacokinetic groups was 99% (115/116). Overall, 179 (76%) patients underwent regular dialysis. The SVR12 rate in the deferred treatment group was 98% (97/99). There were no patients in the immediate and pharmacokinetic group and five (4%) in the deferred treatment group who discontinued treatment because of an adverse event. The most frequent adverse events were nausea, headache, and fatigue—these occurred with similar rates in the three cohorts. One SAE occurred during deferred treatment (interstitial nephritis) and one occurred during the placebo phase of deferred treatment (elevation of lipase levels); these were considered related to DAAs. The investigators observed four deaths, one occurred in the immediate treatment group (cardiac arrest) and three in the deferred treatment group (aortic aneurysm, pneumonia, and unknown cause); the four events were evaluated unrelated to study drugs. No differences occurred in biochemical liver tests between the deferred and immediate treatment groups. All patients enrolled in the C-SURFER study had HCV genotype 1; there were no patients with HCV genotype 4. It is likely that the efficacy of GRZ/EBR in patients with intact kidneys and HCV genotype 1 and 4 can be extrapolated to uremic patients with genotype HCV 4. On the grounds of these data, daily fixed-dose GRZ/EBR is recommended for patients with advanced CKD and HCV genotype 1 and 4.

Another combination of PI containing SOF-free DAAs for treatment of HCV and advanced CKD is glecaprevir (NS3/NS4A protease inhibitor) and pibrentasvir (NS5A inhibitor) for genotype HCV 1, 2, 3, 4, 5 or 6 infection. EXPEDITION-4 was an additional registration trial of DAAs in stage 4–5 CKD [25]. 104 patients were included in a multicentre, open-label phase 3 study (EXPEDITION-4), 82% were haemodialysis-dependent. ITT and mITT SVR12 rates were 98% and 100%, respectively; two patients did not reach SVR12. Adverse events included nausea (12%), fatigue (14%), and pruritus (20%). Serious AEs were observed in 24% of patients (25 of 104); no SAEs related to the study drugs and no viral failures were observed. Two patients did not obtain SVR12—one patient interrupted antiviral treatment due to diarrhoea (and gastrointestinal bleeding) and another cerebral haemorrhage (and uncontrolled hypertension). Patients in the EXPEDITION-4 study received three tablets once daily for 12 weeks, each tablet containing glecaprevir (100 mg) and pibrentasvir (40 mg) [25].

EXPEDITION-5 is a phase 3 study aimed to assess efficacy and safety of the fixed-dose combination of glecaprevir/pibrentasvir for chronic HCV (HCV genotype 1 through 6) in adults without decompensated cirrhosis and with renal insufficiency (Table 2). Duration of therapy with G/P (8, 12, or 16 weeks) was established according to HCV genotype, cirrhosis status and prior treatment experience. Kidney insufficiency was defined as eGFR less than 45 mL/min/1.73 m^2^ (stage 3b, 4, or 5 chronic kidney disease). 101 patients were enrolled in the study, 76% receiving dialysis. Most of the participants received glecaprevir/pibrentasvir for 8 weeks. Overall, the great majority of the participants receiving G/P obtained SVR12, 97% (98/101) 95% CI, 91.6–99); serious AEs occurred in 12% of patients, and none of these were considered to be related to the drug. The conclusion of the investigators was that it is possible to obtain excellent outcomes even with the shorter 8-week regimen of G/P [26].

Additional clinical trials on PI-containing SOF-free DAAs have been published and regarded ritonavir-boosted paritaprevir, ombitasvir, and dasabuvir (RUBY-I and RUBY II) [27,28,29].

## 9. PI-Containing SOF-Free DAAs (Real World Studies)

One major limitation of the information reported above is that it is mostly based on industry-funded studies, and these kinds of studies are more likely to be published if results are favourable [30]. However, numerous real-life studies on the same point have been completed supporting the conclusions reported above [31,32,33,34,35,36,37,38,39,40,41,42,43,44,45].

## 10. SOF-Based DAAs (Clinical Trials)

Some post marketing studies have been published on the antiviral combination sofosbuvir (NS5B inhibitor)/velpatasvir (NS5A inhibitor), which provides an option for HCV-infected patients, regardless of HCV genotype. Borgia and colleagues conducted a phase 2, single-arm multicenter study, and enrolled 59 patients with HCV genotype 1–6 on regular dialysis who received sofosbuvir/velpatasvir. There were patients on haemodialysis (*n* = 54) or peritoneal dialysis (*n* = 5) [46]. Patients underwent long-term dialysis in 22 sites (Canada, UK, Spain, Israel, New Zealand and Australia). Some (*n* = 13, 22%) were treatment-experienced patients. Overall, 56 of 59 (95%) achieved SVR12 after receiving once-daily tablets containing sofosbuvir (400 mg)/velpatasvir (100 mg) for 12 weeks. Serious AEs were reported for 11 patients (19%), and all were considered to be unrelated to DAAS. The most common AEs were headache (*n* = 10, 17%), fatigue (*n* = 8, 14%), nausea (*n* = 8, 14%) and vomiting (*n* = 8, 14%). The study was conducted with aid provided by Gilead Sciences.

Lawitz and coworkers evaluated the efficacy and safety of sofosbuvir with ribavirin or ledipasvir, plus sofosbuvir in patients with CKD (pre-dialysis stage). They conducted a phase 2b, open-label, multicentre study (USA and New Zealand) investigating three sequentially enrolled cohorts of patients [47]. Funding was provided by Gilead Sciences. Only patients with creatinine clearance equal to or less than 30 mL/min, not yet on dialysis, and having HCV genotypes 1 or 3, were enrolled. Cohort 1 patients received 200 mg sofosbuvir plus ribavirin 200 mg once per day for 24 weeks, cohort 2 patients received identical DAAs but greater sofosbuvir dose (400 mg). Cohort 3 patients underwent therapy with ledipasvir plus sofosbuvir (90 mg ledipasvir and 400 mg sofosbuvir) once per day for 12 weeks. The frequency of SVR 12 was 40% (4/10) in cohort 1, 60% (6/10) in cohort 2 and 100% (18/18) in cohort 3. SAEs occurred in eight patients, but none was considered treatment related, the most common AEs were headache (21%, 8/31), anaemia (18%, 7/38) and fatigue (16%, 6/38). The conclusion of the authors was that the combination ledipasvir/sofosbuvir is effective and safe in patients with advanced CKD not yet on dialysis. Another clinical trial was provided by Chuang and coworkers [48].

## 11. SOF-Based DAAs (Real World Studies)

Initial studies had not suggested sofosbuvir-based combinations of DAAs in patients with kidney impairment (Table 2). Sofosbuvir is a first-in-class NS5B inhibitor and is currently the backbone of many combinations of DAAs for HCV treatment in patients with intact kidneys. It is now administered at 400 mg/day. Sofosbuvir is metabolized at the intracellular level to form the active metabolite GS-461203, which is in turn dephosphorylated in the inactive compound GS-331007. GS-331007 is mostly cleared by kidneys (around 78% of the administered dose). In patients with mild, moderate and severe kidney insufficiency, GS-331007 AUC values were greater by 55%, 88%, and 451% in comparison with controls. GS-331007 exposure is increased by at least 10 to 20 times in patients with end-stage kidney disease [49].

Systematic reviews of the scientific literature with meta-analyses of clinical studies concerned sofosbuvir therapy in patients with CKD stage 4–5 [50,51]. We identified thirty clinical studies (*n* = 1537 unique patients) published during the 2015–2020 period. The pooled SVR 12 was 0.99 (95% CI, 0.97; 1.0, *I*^2^ = 99.8%). The overall estimate of the frequency of SAEs was 0.09 (95% CI, 0.05; 0.13, *I*^2^ = 84.3%). Common serious AEs were anaemia (*n* = 26, 38%) and lowered eGFR (*n* = 14, 19%). SAEs were more frequent in studies adopting full-dose sofosbuvir (pooled rate of SAEs 0.15, 95% CI, 0.06; 0.25; *I*^2^ = 80.1%) and in those reports which used ribavirin (0.15, 95% CI, 0.07; 0.23, *I*^2^ = 95.8%). Six studies (69 unique patients) gave data on eGFR at baseline and post- antiviral therapy, no consistent changes were observed [50].

Gaur and colleagues [52] provided real world evidence as they retrospectively evaluated all patients with chronic HCV and end-stage kidney disease on maintenance haemodialysis at a tertiary care institute in northern India. There were 31 patients with treatment-naïve HCV who received sofosbuvir/velpatasvir fixed-dose combination, most patients (*n* = 30, 97%) achieved SVR12. No patient reported major adverse events which required hospital admission, interruption of treatment or death during therapy. Dyspepsia occurred in three (10%) patients and one (3%) had headache. Two (7%) patients had reduced haemoglobin concentrations, one required blood transfusions and the other patient received increased doses of erythropoietin. The most important shortcomings of these studies were the limited size of the study groups, and the exclusion of patients with decompensated liver disease. The absence of a control group made difficult to distinguish AEs related to DAAs or other causes (as an example, underlying comorbidities).

Butt and colleagues conducted an observational cohort study by the database of Electronically Retrieved Cohort of HCV Infected Veterans (ERCHIVES); thus, the study was made in the “real life” setting of the Veteran Administration system [53]. The goal of the study was to assess the virological response to some combinations of DAAs with or without ribavirin (*n* = 13,663 patients on sofosbuvir/ledipasvir and *n* = 3961 ritonavir-boosted paritaprevir/ombitasvir/dasabuvir). There were 2281 patients with stage 3 and 257 with stage 4–5 CKD. The SVR12 rates for patients with stage 3 CKD were 97% (1080/1113) among patients on ledipasvir/sofosbuvir and 97.1% (375/386) among those on ledipasvir/sofosbuvir plus ribavirin. For those patients with stage 4–5 CKD, the SVR12 rates were 94% (78/83) and 100% (25/25) on ledipasvir/sofosbuvir and ledipasvir/sofosbuvir plus ribavirin, respectively. A drop in eGFR from baseline greater than 10 mL/min/1.73 m^2^ was found in 33.1% (2751/8303) and 37.8% (1250/3311), among patients with baseline stage 1–2 CKD who received sofosbuvir/ledipasvir with and without ribavirin, respectively. A drop in eGFR from baseline greater than 10 mL/min/1.73m^2^ was noted in 16.5% (235/1427) and 15.9% (77/485) among patients with baseline stage 3 CKD who received sofosbuvir/ledipasvir with and without ribavirin, respectively [53]. Further evidence based on real life studies has been recently established [54,55,56,57,58,59]

## 12. SOF-Based DAAs and Kidney Impairment

Sofosbuvir is administered by oral route. The most important metabolite is GS-331077 which is the pharmacologically inactive nucleoside and is made by dephosphorylation. Following a single 400 mg oral dose of sofosbuvir, 80% is excreted in urine, and 14% in feces. GS-331007 is renally excreted and accumulates 5- to 20-fold in individuals with stage 4–5 CKD (including those on regular dialysis) [60]. It remained unclear if accumulation of sofosbuvir metabolites was associated with toxicity. Initial studies on sofosbuvir did not include patients with advanced CKD; thus, SOF was not initially licensed for patients with advanced CKD. Some clinical trials regarding efficacy and safety of SOF-based therapies for HCV in patients with end-stage kidney disease (including those on regular dialysis) were conducted and these revealed good results—deterioration of GFR and cardiac adverse events were uncommon. Desnoyer and colleagues [61] performed a multicentre, prospective and observational study on patients who were treated with sofosbuvir (400 mg) daily (*n* = 7) or three times weekly (*n* = 5) after 4-h haemodialysis. Plasma levels of sofosbuvir were measured with liquid chromatography and mass spectrometry before/after haemodialysis and 1.5 after last sofosbuvir intake. Sofosbuvir or its metabolite GS-331007 did not accumulate between haemodialysis sessions or throughout the treatment course, with both treatment regimens. The investigators concluded that clinical and biological tolerance was satisfactory for all patients.

In November 2019, the FDA approved the use of sofosbuvir-based regimens in patients with advanced chronic kidney disease, including those with an eGFR < 30 mL/min and on maintenance dialysis [62].

## 13. Conclusions and Personal Views

There is now availability of several drugs for treatment of HCV, and these proved to be effective and safe even in patients with advanced CKD including dialysis patients; on the other side, a huge gap between high prevalence rates and low treatment coverage within dialysis facilities still exists [63]. The World Health Organization aims to obtain 90% reduction in the incidence of new viral hepatitis infections and 65% reduction in mortality by 2030 [64]. We need to overcome the current barriers for the treatment of HCV in dialysis patients. DAAs are expensive, many countries have restricted criteria of treatment coverage of DAAs, clinicians and/or patients can lack of awareness and motivation, the so-called silo effect occurs (in other words, the reluctance of employees to integrate their efforts within an organization), and difficulties in diagnostic laboratory support (HCV RNA testing) may exist. Finally, nephrologists are not permitted to prescribe DAAs. Studies are being finalized to understand whether successful antiviral therapy with DAAs will improve survival of patients with advanced CKD are under way.

## Figures and Tables

**Table 1:** DAAs, respective targets and advanced chronic kidney disease.

Group	Target	Drug	Others
Protease inhibitors	NS3/NS4A	Simeprevir Paritaprevir Grazoprevir Voxilaprevir Glecaprevir	No dose adjustment in patients with stage 4–5 CKD
Non-nucleoside polymerase inhibitors	NS5B	Dasabuvir Beclabuvir	No dose adjustment in patients with stage 4–5 CKD
Nucleoside polymerase inhibitors	NS5B	Sofosbuvir	Licensed for patients with stage 4–5 CKD (since Nov 2019)
NS5A inhibitors	NS5A	Daclatasvir Elbasvir Ombitasvir Velpatasvir Ledipasvir	No dose adjustment in patients with stage 4–5 CKD

**Table 2:** DAAs in advanced chronic kidney disease: recommended combinations.

DAA Regimen	Dose	HCV Genotype	ClinicalTrials.gov Number (Gov Identifier)
Elbasvir/Grazoprevir	Daily fixed-dose combination (50 mg/100 mg) for 12 weeks	1, and 4	[C-SURFER] NCT 02092350
Glecaprevir/Pibrentasvir	Daily fixed-dose combination (100 mg/40 mg × 3) for 12 or 8 weeks	1, 2, 3, 4, 5, and 6	[EXPEDITION-4] NCT 02651194 [EXPEDITION-5] NCT 03069365
Ledipasvir/Sofosbuvir	Daily fixed-dose combination (90 mg/400 mg) for 12 weeks	1, 4, 5, and 6	NCT 03036852
Sofosbuvir/Velpatasvir	Daily fixed-dose combination (400 mg/100 mg) for 12 weeks	1, 2, 3, 4, 5, and 6	NCT 01958281

## Data Availability

Not applicable.
